# Supplementary material for: The Challenges for EU User Testing Policies for Patient Information Leaflets
Source: Int J Environ Res Public Health. 2024 Sep 29;21(10):1301. doi: 10.3390/ijerph21101301 (PMC11507276; doi:10.3390/ijerph21101301)
Supplement: Supplementary file 1 [file ijerph-21-01301-s001.zip › Supplementary file S1.pdf]

EUROPEAN COMMISSION
ENTERPRISE AND INDUSTRY DIRECTORATE-GENERAL

Consumer goods
Pharmaceuticals

Guidance concerning consultations with target patient groups for the package leaflet

Article 59(3) and 61(1) of Directive 2001/83/EC as amended by Directive 2004/27/EC

1. INTRODUCTION

According to Articles 59(3) and 61(1) of Directive 2001/83/EC as amended by Directive 2004/27/EC new requirements apply to the package leaflet. Article 59(3) as amended requires that consultation with target patient groups ('user consultation') be carried out to demonstrate the readability and usefulness of the package leaflet to patients.
Article 59(3) reads:

> *"The package leaflet shall reflect the results of consultations with target patient groups to ensure that it is legible, clear and easy to use."*

Article 61(1) states that

> "*The results of assessments carried out in cooperation with target patient groups shall also be provided to the competent authority.*"

Article 63(2) states that:

> *"The package leaflet must be written and designed to be clear and understandable, enabling the user to act appropriately"*

In addition Article 28(2) and (3) of Directive 2001/83/EC requires that products authorised through the mutual recognition and decentralised procedures will result in a harmonised package leaflet between Member States.

2. SCOPE

For all marketing authorisations granted after 30 October 2005, all the requirements set out in Directive 2001/83/EC as amended apply. Therefore all package leaflets included in Community or national marketing authorisations have to be checked accordingly and the information about the patient consultation must be included in the application dossier. Further guidance is given in section 8 of this guideline.
For changes to existing marketing authorisations, the need for user consultation covers in principle situations where significant changes are made to the package leaflet, either through a variation or a procedure according to Article 61(3) of Directive 2001/83/EC.

3. FORMS OF PATIENTS CONSULTATION

Articles 59(3) and 61(1) of Directive 2001/83 require that the package leaflet shall reflect the results of consultations with target patient groups to ensure that it is legible, clear and easy to use and that these results of assessments carried out in cooperation with target patient groups shall also be provided to the competent authority.

They do not define the precise method to be used. As a consequence, these provisions permit user testing as well as other appropriate forms of consultation.

3.1 User Testing

One of the possible ways of complying with the new legal requirement is by performing a 'user testing' of the package leaflet.
User testing means to test the readability of a specimen with a group of selected test subjects. It is a development tool which is flexible and aims to identify whether or not the information as presented, conveys the correct messages to those who read it. Testing itself does not improve the quality of the information but it will indicate where there are problem areas

which should be rectified. The user testing should be part of Module 1 of the application dossier.

3.2 Other methods

Other methods than user testing may be acceptable provided that the outcome ensures that the information is legible, clear and easy to use so that patients can locate important information within the package leaflet, understand it and enables the user to act appropriately. Such alternative methodology will have to be justified by the applicant/marketing authorisation holder and will be considered on a case-by-case basis.

4. DEMONSTRATION OF PATIENTS CONSULTATION

In general, performing the user testing or another justified consultation method will be essential prior to granting or varying any marketing authorisation under either the centralised, mutual recognition, decentralised or national procedures.

Member States and the European Medicines Agency agreed on harmonised Quality Review of Documents (QRD) templates for the package leaflet to ensure that the statutory information appears as intended by the Directive 2001/83/EC as amended. Compliance with the QRD templates does not exempt from the obligation to undertake a user test or other form of user consultation.

a) New consultation for a medicinal product

In the following situations a user consultation is always required: First authorisation of a medicinal product with a new active substance,

- Medicinal products which have undergone a change in legal status,
- Medicinal products with a new presentation,
- Medicinal products with particular critical safety issues.

b) Reference to already approved package leaflets according to Article 59(3) and Article 61(1) of Directive 2001/83/EC

The evidence from tests on similar package leaflets may be used where appropriate. Examples of when this may be considered acceptable based on a sound justification by the applicant/marketing authorisation holder are:

- extensions for the same route of administration e.g. intravenous/intramuscular or oropharyngeal/laryngopharyngea,
- same safety issues identified,
- same class of medicinal product.

It may be appropriate for an applicant/marketing authorisation holder to refer to a representative sample of package leaflets for medicinal products which comply with the new legislative requirements. The types of package leaflets should be chosen carefully to be representative of one or more of the following considerations:

- recently approved package leaflets for a corresponding medicinal product,
- reflect complex issues of risk communication which may need careful handling,
- medical terminology which requires detailed explanation .

However, certain package leaflets may require further user consultation to provide reassurance that patients will benefit from the information provided. This is e.g. the case where user consultation concentrates on one particular aspect of a leaflet which may need particular patient attention, e.g. expression of risk of side effects or complex instructions how to administer the medicinal product.

5. TESTING OF MULTIPLE LANGUAGE VERSIONS

The package leaflet should be legible, clear and easy to read in all EEA languages. As a matter of principle it is normally sufficient to undertake patient consultation in one EEA language. Results of such consultation should be presented in English for the centralised, decentralised and mutual recognition procedure, or in the national language for national procedures to permit the assessment of the test to be undertaken by competent authority responsible for granting the marketing authorisation.
In the centralised, decentralised and mutual recognition procedure, only the English language version of the package leaflet will be agreed during the scientific assessment.

The quality of translation should be the focus of a thorough review by the applicant/marketing authorisation holder once the original package leaflet has been properly tested and modified.

During the drafting of the original package leaflet every effort should be made to ensure that the package leaflet can be translated from the original to the various national languages in a clear and understandable way. It is important that the outcome of the user consultation is then correctly translated into the other languages. Strict literal translations from the original language may lead to package leaflets which contain unnatural phrases resulting in a package leaflet which is difficult for patients to understand. Therefore, different language versions of the same package leaflet should be 'faithful' translations allowing for regional translation flexibility, whilst maintaining the same core meaning.

Following the grant of the marketing authorisation, the responsibility for the production of faithful translations will rest with the marketing authorisation holder in consultation with the Member States/European Medicines Agency.

If user consultation has been performed on a package leaflet in the old QRD template, there is no need to be retested when updating according to the new QRD template.

6. PRESENTATION OF RESULTS

The presentation of results should be shortened to a summary explaining how the consultation was executed and how the resulting package leaflet accommodated any need for change. The summary should be in Module 1.3.4 of the application and should have the following structure:

1. Product description
2. Consultation or test details, such as:
   - Method used
   - Explanation on the choice of population consulted
   - Language(s) tested
3. Questionnaire (including instructions and observation forms)

4. Original and revised package leaflets
5. Summary and discussion of results (subjects' answers, problems identified and revisions made to relevant package leaflet section)
6. Conclusion

All other details should be available on demand.
The report and the results of the consultation should be presented in English for the centralised, decentralised and mutual recognition procedure or in the national language for national procedures.

7. APPROVAL BY THE COMPETENT AUTHORITY

In approving package leaflets the competent authorities will look for evidence that people who are likely to rely on the package leaflet can understand it and act appropriately. Any consultation submitted in support of a package leaflet will need to cover the following:

- Data gathered from users under defined conditions
- The people who are likely to rely on the package leaflet for a particular medicine will depend upon a number of factors and may include carers (e.g. parents, partners, friends, as well as nursing assistants) rather than patients if the medicine is generally intended for administration by someone other than the patient.
- In order to ensure that those involved can understand and apply the information, the evidence presented must demonstrate that they can pick out the relevant information, interpret this and describe the action they would take as a result.
- The key information will need to be defined prior to the consultation by the marketing authorisation holder and is likely to include significant side effects, warnings, what the medicine is for and how to take/use the product.

8. OTHER ISSUES FOR CONSIDERATION

The Member States or the European Medicines Agency will have considered other aspects in relation to consultation or user testing and usability of package leaflets and additional guidance is available or under development concerning:

- Timing of user consultation, submission and assessment within the evaluation procedure;
- Guidance in relation to usability and presentation of information;
- Guidance on how user testing should be carried out and what alternative methods are acceptable.
