# Supplementary material for: The Challenges for EU User Testing Policies for Patient Information Leaflets
Source: Int J Environ Res Public Health. 2024 Sep 29;21(10):1301. doi: 10.3390/ijerph21101301 (PMC11507276; doi:10.3390/ijerph21101301)
Supplement: Supplementary file 1 [file ijerph-21-01301-s001.zip › Supplementary file S2.pdf]

EUROPEAN COMMISSION
ENTERPRISE AND INDUSTRY DIRECTORATE-GENERAL

Consumer goods
Pharmaceuticals

GUIDELINE ON THE READABILITY OF THE LABELLING AND PACKAGE LEAFLET OF MEDICINAL PRODUCTS FOR HUMAN USE

Keywords: Label, package leaflet, medicinal products for human use, readability

TABLE OF CONTENTS

LEGAL FRAMEWORK 5

PURPOSE OF THIS GUIDELINE 6

CHAPTER 1 READABILITY OF THE PACKAGE LEAFLET AND THE LABELLING 7

SECTION A RECOMMENDATIONS FOR THE PACKAGE LEAFLET 7

GENERAL CONSIDERATIONS 7

1. TYPE SIZE AND FONT 7

2. DESIGN AND LAYOUT OF THE INFORMATION 8

3. HEADINGS 8

4. PRINT COLOUR 8

5. SYNTAX 9

6. STYLE 9

7. PAPER 10

8. USE OF SYMBOLS AND PICTOGRAMS 10

9. ADDITIONAL INFORMATION 10

9.1. Product ranges 10

9.2. Products administered by a healthcare professional or in a hospital 11

10. TEMPLATES FOR THE PACKAGE LEAFLET 11

SECTION B RECOMMENDATIONS FOR THE LABELLING 12

GENERAL CONSIDERATIONS 12

1. NAME OF THE MEDICINE 12

2. STRENGTH AND TOTAL CONTENT 13

3. ROUTE OF ADMINISTRATION 13

4. DESIGN AND LAYOUT 13

5. TEMPLATES FOR LABELLING 14

6. OTHER INFORMATION 15

7. BLISTER PACK PRESENTATIONS 15

8 SMALL CONTAINERS 15

CHAPTER 2 SPECIFIC RECOMMENDATIONS FOR BLIND AND PARTIALLY- SIGHTED PATIENTS 16

1. LEGAL TEXT 16

2. IMPLEMENTATION 16

3. BRAILLE 16

4. SCOPE 17

5. PACKAGING 18

6. PACKAGE INFORMATION LEAFLET FOR BLIND AND PARTIALLY-SIGHTED 18

CHAPTER 3 GUIDANCE CONCERNING CONSULTATIONS WITH TARGET PATIENT GROUPS FOR THE PACKAGE LEAFLET 19

1. INTRODUCTION 19

2. SCOPE 19

3. FORMS OF PATIENT CONSULTATION 19

3.1 User testing 20

3.2 Other methods 20

4. DEMONSTRATION OF PATIENT CONSULTATIONS 20

4.1 New consultation for a medicinal product 20

4.2 Reference to already approved package leaflets according to Article 59(3) and Article 61(1) of Directive 2001/83/EC 21

5. TESTING OF MULTIPLE LANGUAGE VERSIONS 21

6. PRESENTATION OF RESULTS 22

7. APPROVAL BY THE COMPETENT AUTHORITY 23

ANNEX - ILLUSTRATION - ONE WAY OF UNDERTAKING A TEST OF A PACKAGE LEAFLET 24

1. PERFORMING THE TEST 24

2. RECRUITING PARTICIPANTS 24

3. SUGGESTED TESTING PROCEDURE 25

4. PREPARING FOR THE TEST 25

5. SUCCESS CRITERIA 26

Legal framework

All medicinal products placed on the Community market are required by Community law to be accompanied by labelling and package leaflet which provide a set of comprehensible information enabling the use of the medicinal product safely and appropriately.

According to Article 54, Article 55 and Article 59 of Directive 2001/83/EC of the European Parliament and of the Council of 6 November 2001 on the Community code relating to medicinal products for human use[1] (hereinafter: "**Directive 2001/83/EC**") medicinal products must be accompanied by outer and/or immediate packaging information (labelling) and a package leaflet.

Article 58 of Directive 2001/83/EC allows for the omission of a package leaflet where all the required information can be directly conveyed on the packaging.

Article 56 of Directive 2001/83/EC requires that the particulars to be included in the labelling shall be easily legible, clearly comprehensible and indelible.

Article 56a of Directive 2001/83/EC requires the name of the medicinal product (as referred to in Article 54(a)) to be expressed in Braille format on the packaging, and the marketing authorisation holder to ensure that the package leaflet is made available on request from patients' organisations in formats appropriate for the blind and partially- sighted.

Article 59(3) of Directive 2001/83/EC provides that the package leaflet shall reflect the results of consultations with target patient groups to ensure that it is legible, clear and easy to use.

Articles 61(1) and 8(3)(j) of Directive 2001/83/EC specify that one or more mock-ups of the outer packaging and the immediate packaging of a medicinal product, together with the draft package leaflet, shall be submitted to the competent authority at the time of marketing authorisation application. The results of assessments carried out in cooperation with target patient groups shall also be provided.

Article 63(1) of Directive 2001/83/EC requires that the labelling and package leaflet shall appear in the official language or languages of the Member State where the product is placed on the market. Additional languages can be included provided the information presented is the same in all languages.

Article 63(2) of Directive 2001/83/EC requires that the package leaflet must be written and designed to be clear and understandable, enabling the users to act appropriately, when necessary with the help of health professionals. The package leaflet must be clearly legible in the official language or languages of the Member State(s) in which the medicinal product is placed on the market.

---

1 OJ L 311, 28.11.2001, p. 67.

Purpose of this guideline

The main purpose of this document is to provide guidance on how to ensure that the information on the labelling and package leaflet is accessible to and can be understood by those who receive it, so that they can use their medicine safely and appropriately.

This guideline is written to assist applicants and marketing authorisations holders when drawing up the labelling and package leaflet and preparing the mock-ups or specimens of the sales presentations[2].

The guidance gives advice on the presentation of the content of the labelling and package leaflet (required in accordance with Title V of the Directive) and on the design and layout concepts which will aid the production of quality information. It includes guidance on consultations with target patient groups for the package leaflet.

The guideline also includes information on how the requirements for Braille can be met, as well as how to make the package leaflet available in formats suitable for the blind and partially-sighted patients.

Finally, the guideline includes an example of a way of undertaking a test of a package leaflet.

This guideline is published in accordance with Article 65(c) of Directive 2001/83/EC, which provides for the development of guidelines concerning the legibility of particulars on the labelling and package leaflet.

The guideline is intended to apply to all marketing authorisation procedures and to all medicinal products, including those available without prescription.

---

[2] A mock-up is a copy of the flat artwork design in full colour, presented so that, following cutting and folding where necessary, it provides a replica of both the outer and immediate packaging so that the three dimensional presentation of the labelling text is clear. This mock-up is generally referred to as a paper copy and not necessarily in the material of the sales presentation. A specimen is a sample of the actual printed out outer and immediate packaging materials and package leaflet (i.e. the sales presentation).

Chapter 1 Readability of the package leaflet and the labelling

SECTION A RECOMMENDATIONS FOR THE PACKAGE LEAFLET

GENERAL CONSIDERATIONS

The package leaflet is intended for the patient/user. If the package leaflet is well designed and clearly worded, this maximises the number of people who can use the information, including older children and adolescents, those with poor literacy skills and those with some degree of sight loss. Companies are encouraged to seek advice from specialists in information design when devising their house style for the package leaflet to ensure that the design facilitates navigation and access to information.

The following guidance sets out recommendations on various aspects related to the preparation of package leaflets. It is aimed at helping applicants/marketing authorisation holders to fully comply with the legal requirements and is based on experience where it has been shown that using these techniques optimises the usability of the package leaflet.

Additional requirements may apply in particular Member States. Applicants should check details of those requirements in the Notice to Applicants, Volume 2A, chapter 7.

1. TYPE SIZE AND FONT

Choose a font which is easy to read. Stylised fonts which are difficult to read should not be used. It is important to choose a font in which similar letters/numbers, such as “i”, “l” and “1” can be easily distinguished from each other.

The type size should be as large as possible to aid readers. A type size of 9 points, as measured in font ‘Times New Roman’, not narrowed, with a space between lines of at least 3 mm, should be considered as a minimum. However, for marketing authorisation applications until 1 February 2011, a type size of 8 points, as measured in font ‘Times New Roman’, not narrowed, with a space between lines of at least 3 mm, should be acceptable as absolute minimum.

Consideration should be given to using different text sizes to enable key information to stand out and to facilitate navigation in the text (for example, for headings).

Consideration should be given to using larger type size where a medicinal product is especially intended for an indication linked to visual impairment (see also Chapter 2 section 6).

The widespread use of capitals should not be used. The brain recognises words in written documents by the word shape, so choose lower case text for large blocks of text. However, capitals may be useful for emphasis.

Do not use italics and underlining as they make it more difficult for the reader to recognise the word-shape. Italics, however, may be considered when using Latin terms.

2. Design and layout of the information

The use of “justified” text (that is text aligned to both left hand and right hand margins) should in principle not be used.

Line spaces should be kept clear. The space between lines is an important factor influencing the clarity of the text. As a general rule the space between one line and the next should be at least 1.5 times the space between words on a line, where practical.

Contrast between the text and the background is important. Factors like paper weight, colour of the paper, size and weight of the type, colour of the type and the paper itself should be considered. Too little contrast between the text and the background adversely affects the accessibility of the information. Therefore, background images should in principle not be placed behind the text since they may interfere with the clarity of the information making it harder to read.

A column format for the text can help the reader navigate the information. The margin between the columns should be large enough to adequately separate the text. If space is limited a vertical line to separate the text may be used. Related information should be kept together so the text flows easily from one column to the next. Consideration should be given to using a landscape layout which can be helpful to patients. Where a multi- lingual leaflet is proposed there should be a clear demarcation between the different languages used; all the information provided in each language should be assembled.

3. Headings

Headings are important and can help patients navigate the text if used well. Therefore, bold type face for the heading or a different colour, may help make this information standout. The spacing above and below the headings should be consistently applied throughoutthe leaflet. Same level headings should appear consistently (numbering, bulleting, colour, indentation, font and size) to aid the reader.

The use of multiple levels of headings should be considered carefully, as more than two levels may make it difficult for readers to find their way around the leaflet. However, where complex information has to be communicated multiple levels of headings may be needed.

Using lines to separate the different sections within the text can also be helpful as a navigational tool.

Include all main section headings covered by Article 59(1) of Directive 2001/83/EC within the leaflet. Sub-headings and associated text within the leaflet should only be included if these are relevant for the particular medicine. For example if there is no information in relation to excipients of known effect this section may be omitted from the package leaflet.

4. Print colour

Accessibility is not only determined by print size. Characters may be printed in one or several colours allowing them to be clearly distinguished from the background. A different type size or colour is one way of making headings or other important information clearly recognisable.

The relationship between the colours used is as important as the colours themselves. As a general rule dark text should be printed on a light background. But there may be occasions when reverse type (light text on a dark background) could be considered to highlight for instance particular warnings. In such circumstances the quality of the print will need careful consideration and may require the use of a larger type size or bold text. Similar colours should not be used for the text and background as legibility is impaired.

5. SYNTAX

Some people may have poor reading skills, and some may have poor health literacy. Aim to use simple words of few syllables.

Long sentences should not be used. It is better to use a couple of sentences rather than one longer sentence, especially for new information.

Long paragraphs can confuse readers, particularly where lists of side effects are included. The use of bullet points for such lists is considered more appropriate. Where possible, no more than five or six bullet points in a list are recommended.

When setting out the side effects it is particularly important to consider the order in which they are given so the patients/users may maximise the use of the information. In general, setting out the side effects by frequency of occurrence, starting with the highest frequency, is recommended to help communicate the level of risk to individuals. Frequency terms should be explained in a way patients/users can understand – for example"very common" (more than 1 in 10 patients). However, where a serious side effect exists which would require the patient/user to take urgent action this should be afforded greater prominence and appear at the start of the section. Setting side effects by organ/system/class is not recommended since patients/users are in general not familiar with these classifications.

6. STYLE

When writing, an active style should be used, instead of passive. For example:

- '*take 2 tablets'* instead of *'2 tablet should be taken','*
- '*you must....'* is better than *'it is necessary ...*'

When telling patients what action to take, reasons should be provided. Instructions should come first, followed by the reasoning, for example: 'take care with X if you have asthma – it may bring on an attack'.

"Your medicine, this medicine, etc." should be used rather than repeating the name of the product, as long as the context makes clear what is being referred to.

Abbreviations and acronyms should not usually be used unless these are appropriate. When first used in the text, the meaning should be spelled out in full. Similarly scientific symbols (e.g. > or <) are not well understood and should not be used.

Medical terms should be translated into language which patients can understand. Consistency should be assured in how translations are explained by giving the lay term

with a description first and the detailed medical term immediately after. On a case by case basis the most appropriate term (lay or medical) may then be used thereafter throughout the package leaflet in order to achieve a readable text. Make sure that the language used alerts the reader to all the information relevant to him/her, and gives sufficient detail on how to recognise possible side effects and understand any action which may be necessary.

7. PAPER

The paper weight chosen should be such that the paper is sufficiently thick to reduce transparency which makes reading difficult, particularly where the text size is small. Glossy paper reflects light making the information difficult to read, so the use of uncoated paper should be considered.

Make sure that when the leaflet is folded the creases do not interfere with the readability of the information.

8. USE OF SYMBOLS AND PICTOGRAMS

The legal provisions within Article 62 of Directive 2001/83/EC permit the use of images, pictograms and other graphics to aid comprehension of the information, but these exclude any element of a promotional nature. Symbols and pictograms can be useful provided the meaning of the symbol is clear and the size of the graphic makes it easily legible. They should only be used to aid navigation, clarify or highlight certain aspects of the text and should not replace the actual text. Evidence may be required to ensure that their meaning is generally understood and not misleading or confusing. If there is any doubt about the meaning of a particular pictogram it will be considered inappropriate. Particular care will be needed when symbols are transferred or used in other language versions of the leaflet and further user testing of these may be necessary.

9. ADDITIONAL INFORMATION

9.1. Product ranges

There should, in principle, be a separate leaflet for each strength and pharmaceutical form of a medicinal product. On a case-by-case basis national competent authorities or the European Commission may however agree to allow the use of combined package leaflets for different strengths and/or different pharmaceutical forms (e.g. tablets and capsules), for instance where achieving a recommended dose necessitates a combination of different strengths, or when the dose varies from day to day depending on the clinical response.[3]

Simple reference to other strengths and pharmaceutical forms of the same medicine is always possible if necessary for the therapy. For instance, referring to a different strength,

---

[3] Concerning combined package leaflets for different strengths for medicines authorised through the centralised procedure, applicants may wish to consult guidance provided by the EMEA at http://www.emea.europa.eu/htms/human/qrd/qrdplt/2509002.pdf.

or referring in the package leaflet of a tablet which is unsuitable for children to the availability of an oral solution for children.

9.2. Products administered by a healthcare professional or in a hospital

For a product administered by a healthcare professional, information from the summary of product characteristics for the healthcare professional (e.g. the instructions for use) could be included at the end of the patient leaflet e.g. in a tear-off portion, to be removed prior to giving the leaflet to the patient. Alternatively the complete summary of product characteristics could be provided in the pack along with the package leaflet.

For a product administered in hospital additional package leaflets (in addition to the one provided in the pack) may be made available on request to ensure that every patient receiving the medicine has access to the information.

10. TEMPLATES FOR THE PACKAGE LEAFLET

The templates provided in all EEA languages on the EMEA Website (http://www.emea.europa.eu/htms/human/qrd/qrdtemplate.htm) reflect the particulars which must appear on the labelling and package leaflet of medicinal products according to Directive 2001/83/EC. They will help to ensure that the information appears as intended by the Directive, and to ensure consistency in the information provided across a number of different medicines and across Member States.

For the purpose of regulatory submissions to national competent authorities/EMEA, the text version of the product information is to be presented in the format and lay-out (see "QRD convention" on the EMEA Website at http://www.emea.europa.eu/htms/human/qrd/qrdplt/qrdconvention.pdf) using the electronic product information templates.

When using these templates, reference should be made to relevant Community Guidelines, QRD Guidance and the "Annotated QRD Template", which provides detailed guidance on how to complete each section and which can be found on the EMEA Website (http://www.emea.europa.eu/htms/human/qrd/qrdplt/AnnotatedTemplate-H.pdf) and the Heads of Agencies Website (http://www.hma.eu/uploads/media/QRD_annotated_template_CMDh.pdf).

Having used the templates provided, marketing authorisation applicants/holders will still need to format the resulting text into the relevant full colour mock-ups or specimens of the package leaflet. Also applicants should remember that using the template does not guarantee compliance with Article 59(3) of the directive and consultations with target patient groups will still have to be carried out on the full colour mock-up or specimen of the package leaflet.

SECTION B RECOMMENDATIONS FOR THE LABELLING

GENERAL CONSIDERATIONS

Labelling covers both outer packaging and inner packaging. Although inner packaging may include a lesser set of particulars, many of the principles outlined in relation to outer packaging will apply equally to the labelling of blister packs or other small package units.

Labelling ensures that the critical information necessary for the safe use of the medicine is legible, easily accessible and that users of medicines are assisted in assimilating this information so that confusion and error are minimised.

Those involved in the design of labelling should consider the following sections prior to submission to the competent authority. The recommendations given in relation to the package leaflet (section A) may be applicable to labelling and should be borne in mind in designing and laying out the required information on labels. The particulars appearing on the label of all medicinal products should be printed in characters of at least 7 points (or of a size where the lower case "x" is at least 1.4 mm in height), leaving a space between lines of at least 3 mm.

In particular the information presented on small packs will need careful consideration so that the text is presented in as large a type size as possible to reduce the likelihood of medication error.

According to Article 57 of Directive 2001/83/EC, additional labelling requirements may apply in particular Member States in respect of price, reimbursement conditions, legal status for supply and identification and authenticity. Applicants should check details of those requirements in the Notice to Applicants, Volume 2A, chapter 7.

Labelling must contain all elements required by Article 54 of Directive 2001/83/EC or a lesser set of elements where the provisions of Article 55 of the same Directive apply. Nevertheless, of the information items listed in Article 54 of Directive 2001/83/EC, certain items are deemed critical for the safe use of the medicine. These items are:

- name of the medicine;
- strength and, where relevant, total content;
- route of administration.

Where possible these should be brought together using a sufficiently large type size on the labelling. Having these items together in the same field of view should be considered in order to aid users.

1. NAME OF THE MEDICINE

Article 54(a) of Directive 2001/83/EC sets out what is required in relation to the name of the medicinal product. The full name of the medicinal product, with its strength and its pharmaceutical form, and, if appropriate, whether it is intended for babies, children or adults, should appear on the outer packaging and on the immediate packaging to aid accurate identification of the medicinal product.

Where the medicinal product contains up to three active ingredients, the INN/common name(s) of these active ingredient(s) should be stated after the full name on the outer packaging and the immediate packaging, unless the INN/common name(s) is part of the name. The INN should be afforded due prominence for safety reasons. Where space is at premium the shortened term for pharmaceutical form, as stated in the in the list of EDQM "Standard Terms" may be used on small immediate packaging.

For requirements concerning Braille, see Chapter 2.

2. Strength and total content

In some cases the packaging may need to contain information on both the quantity per unit volume and on the total quantity per total volume. The total quantity per total volume can be particularly important for safety reasons for injectable products and other medicines available in solution or suspension.

Different strengths of the same medicinal product should be expressed in the same manner: for example 250 mg, 500 mg, 750 mg, 1000 mg and NOT 1 g. Trailing zeros should not appear (2.5 mg and NOT 2.50 mg). The use of decimal points (or comma) should be avoided where these can be removed (i.e. 250 mg is acceptable whereas 0.25 g is not). For safety reasons it is important that micrograms is spelt out in full and not abbreviated. However, in certain instances where this poses a practical problem which cannot be solved by using a smaller type size then abbreviated forms may be used, if justified and if there are no safety concerns.

3. Route of administration

This should be as registered in the summary of product characteristics (SPC) only according to the standard terms. Negative statements should not be used: for example "Not for intravenous use". In principle only standard abbreviations may be acceptable (i.v., i.m., s.c.,). In addition, a list of other, non-standard abbreviations which can be usedin SPC and labelling is published on the EMEA website (http://www.emea.europa.eu/htms/human/qrd/docs/listnonstandard.pdf). Other non-standard routes of administration should be spelled out in full. Some routes of administration will be unfamiliar to patients and may need to be explained within the package leaflet. This is particularly important when medicinal products are made available for self-medication.

4. Design and layout

Applicants and marketing authorisation holders should make best use of the space available to ensure that the important information is clearly mentioned on prime spaces onthe outer and immediate packaging, presented in a sufficiently large type size. Company logos and pictograms (if accepted in accordance with Article 62) may be presented, wherespace permits, on the outer packaging and on immediate packaging, provided they do not interfere with the legibility of the mandatory information.

Use of a large type size will be appropriate, although other factors may also be important in making the information legible. Consideration should be given to the line-spacing and use of white space to enhance the legibility of the information provided. For some small

packs it may not be possible to present all the critical information in the same field of view. The use of any innovative technique in packaging design to aid in the identificationand selection of the medicinal product is encouraged. It is also encouraged where space isat a premium.

Colours should be chosen to ensure a good contrast between the text and the background to assure maximum legibility and accessibility of the information. Highly glossy, metallic or reflective packaging should be avoided, as this affects the legibility of the information. Different colours in the name of the product are discouraged since they may negatively impact on the correct identification of the product name. The use of different colours to distinguish different strengths is strongly recommended.

Similarity in packaging which contributes to medication error can be reduced by the judicious use of colour on the pack. The number of colours used on packs will need careful consideration as too many colours could confuse. Where colour is used on the outer pack it is recommended that it is carried onto primary packaging to aid identificationof the medicine.

Where a multi-lingual outer and/or immediate packaging is proposed there should be a clear demarcation between different languages where space permits.

All outer packaging must include space for the prescribed dose to be indicated and/or "blue box"[4] information as required by Member States (see section 6)

5. TEMPLATES FOR LABELLING

The templates provided in all EEA languages on the EMEA Website http://www.emea.eu.int/htms/human/qrd/qrdtemplate.htm reflect the particulars which must appear on the labelling and package leaflet of medicinal products according to Directive 2001/83/EC. They will help to ensure that the information appears as intended by the Directive, and to ensure consistency in the information.

For the purpose of regulatory submissions to national competent authorities/EMEA, the text version of the product information is to be presented in the mandatory format and lay-out (see "QRD convention" on the EMEA Website at http://www.emea.europa.eu/htms/human/qrd/qrdplt/qrdconvention.pdf) using the electronic product information templates.

When using these templates, reference should be made to relevant Community Guidelines, QRD Guidance and the "Annotated QRD Template", which provides detailed guidance on how to complete each section and which can be found on the EMEA Website (http://www.emea.europa.eu/htms/human/qrd/qrdplt/AnnotatedTemplate-H.pdf) and the Heads of Agencies Website (http://www.hma.eu/uploads/media/QRD_annotated_template_CMDh.pdf).

Having used the templates provided, marketing authorisation holders will still need to format the resulting text into the relevant full colour mock-ups and specimens of the packaging.

---

[4] For centrally authorised products, the "Blue box" is a boxed area included in the labelling, with a blue border, aimed at containing information specific to each Member State.

6. OTHER INFORMATION

As foreseen by Article 57 of Directive 2001/83/EC, a Member State may ask for additional information to appear on the packaging concerning identification and authenticity of product, the legal category for supply and the price. National rules will apply in these circumstances and details on the requirements for the "blue box" in mutualrecognition and decentralised procedures are given in the Notice to Applicants, Volume 2A, chapter 7. The "blue box" requirements in the centralised procedure are set out in theNotice to Applicants Volume 2C, "Guideline on the packaging information of medicinal products for human use authorised by the Community".

7. BLISTER PACK PRESENTATIONS

For blister pack presentations it is important that the particulars remain available to the user up to the point at which the last dose is removed. Often it will not be possible to apply all the information over each blister pocket, consequently where a random display ofthe information is proposed it should frequently appear across the pack. In all cases it willbe acceptable to apply the batch number and expiry date to the end of the blister strip. If technically possible, applying this information to both ends of each strip should be considered. Where a unit-dose blister presentation is proposed all the information required for blister packs must appear on each unit dose presentation.

In addition, blister foils should be printed to ensure maximum legibility of the information using a sufficiently large font.

Colour for the text and the font style, should be chosen carefully as the legibility of the text on the foil is already impaired due to the nature of the material. Where possible, non-reflective material or coloured foils should be considered to enhance the readability of the information presented and the correct identification of the medicine.

8 SMALL CONTAINERS

Where the labelling particulars set out in article 54 of Directive 2001/83/EC cannot be applied in full to the labelling of small containers, as a minimum the particulars set out in Article 55(3) of the directive should be applied. Other information required in Article 54 may be added as appropriate, where space permits. The criteria for small container status would normally apply to containers of nominal capacity of 10ml or less. However, other factors may need to be taken into account such as the amount of information which has to be included and the font size necessary to ensure the legibility of the information.

Innovative pack design is encouraged where space is at a premium (e.g. the use of wrap-around or concertina labels). Paper labels are recommended to increase the legibility of the information applied to, for example, ampoules.

Chapter 2 Specific recommendations for blind and partially-sighted patients

Directive 2004/27/EC amending Directive 2001/83/EC included changes to the label and package leaflet requirements.

This guidance interprets the requirements for Braille on the packaging, and the requirements for the package leaflet to be made available in formats for the blind and partially-sighted according to Article 56a.

1. Legal text

Directive 2001/83/EC as amended by Directive 2004/27/EC, Article 56(a)

> *"The name of the medicinal product, as referred to in Article 54, point (a) must also be expressed in Braille format on the packaging. The marketing authorization holder shall ensure that the package information leaflet is made available on request from patients' organisations in formats appropriate for the blind and partially-sighted."*

Directive 2001/83/EC as amended by Directive 2004/27/EC, Article 54(a)

> *"The name of the medicinal product, followed by its strength and pharmaceutical form, and if appropriate, whether it is intended for babies, children or adults; where the product contains up to three active substances, the international non-proprietary name (INN) shall be included, or, if one does not exist, the common name."*

2. Implementation

The provision of Article 56a will apply after the end of the implementation period – 30 Oct 2005 – to all medicinal product approved after this date. It will not apply immediatelyto products authorized before 30 October 2005.

Nevertheless companies are encouraged to apply the provision to all medicinal products as soon as possible. For specific implementation requirements reference is made to the relevant national legislation and EMEA guidance for Centrally Authorised Products.

3. Braille

Braille is the internationally widespread reading and writing system for blind and partially-sighted people. The system was founded in 1825 by Louis Braille (1809 –1852), who lived in France and himself was blind. Braille is not a language, it is just another way to read and write a language.

Braille consists of arrangements of dots which make up the letters of the alphabet, numbers and punctuation marks. The basic Braille symbol is called the Braille cell.

Due to the reason that there are differences in Braille in different countries, the type of Braille letter (size of Braille cell) has to be standardized. The use of Marburg Medium is highly recommended.

The uncontracted Braille system should be used. In this system every Braille character (Braille cell) makes up the letter of the alphabet, punctuation mark, numbers, etc. The contracted Braille system with letter-combinations should not be used, except in small volume packaging (up to 10 ml volume) – see paragraph below under "Scope".

4. SCOPE

"The name of the medicinal product, as referred to in Article 54a" should be interpreted in a way which allows clear identification for blind people. According to the definition in Article 1(20) of Directive 2001/83/EC as amended "the name, which may be either an invented name not liable to confusion with the common name, or a common or scientific name accompanied by a trade mark or the name of the marketing authorization holder", the (invented) name of the medicinal product followed by its strength should be put in Braille on the packaging of the product.

For medicinal products authorised only in a single strength, it is acceptable that only the invented name in Braille is put on the packaging.

This interpretation does not prevent companies to express further information (pharmaceutical form, and if appropriate, whether it is intended for babies, children or adults, etc) in Braille on bigger volume packages on a voluntary basis. Also the inclusion of the expiry date in Braille would be welcome, although it is acknowledged that this may not always be feasible.

For Herbal Medicinal Products the Braille requirement will be restricted to the invented name of the Medicinal Product only. Where the name consists of the active substance(s), information could be limited to the plant name (+ plant part in those cases where several parts are available), plus the type of preparation and the strength in those cases where several strengths exist.

In case of small volume packages (up to 10 ml) with limited space capacity, alternative means of providing Braille information may be considered, eg. use of contracted Braille system or certain defined abbreviations or addition of supplementary "tab" label. Particular consideration should be given to medicinal products likely to be used by a highvisually impaired target population, eg. certain eye drop preparations.

In case of multilingual packaging, the name in Braille has to be printed in all the different languages concerned. Companies are encouraged to use the same invented name for the same medicinal product.

There is no need to put the name in Braille on the packaging of products which are only intended for administration by health care professionals, for example it is not required to put the name in Braille for vaccines.

5. PACKAGING

The name in Braille does not have to be printed on the immediate packaging - such as blisters, ampoules and bottles it only has to appear on the outer/secondary packaging, which is normally a carton. In case where there is no secondary packaging, e.g. large volume bottles (500 ml, 1000 ml, etc.), it is possible to fix an adhesive Braille label around the bottle during the manufacturing process.

On a voluntary basis companies can put the name in Braille on all packaging components.

Affixing an adhesive Braille label at the point of sale/dispensing of the medicinal product on request is not recommended, due to the risk of affixing the wrong Braille label and confusion.

Concerning the location of the Braille on the outer packaging there is no need to put the Braille dots on an empty space of the packaging, but the underlying printed text has to be easily legible.

Where Braille is present on the (outer) packaging of a medicinal product, parallel importer/parallel distributor should ensure that the same Braille text is provided in the language(s) of the member state of destination and that the original Braille text will not cause confusion.

6. PACKAGE INFORMATION LEAFLET FOR BLIND AND PARTIALLY-SIGHTED

On request from patients' organisations the package leaflet should be provided for partially-sighted people in a suitable print, taking into consideration all aspects determining the readability (eg. fontsize: Sans serif typefaces, 16 - 20 point, contrast: black letters on white paper, word spacing, text alignment, line spacing, layout, paper quality). For blind people the text has to be provided in an appropriate format, it is recommended to provide the text in a format perceptible by hearing (CD-ROM, audiocassette, etc.). In certain cases the appropriate format may be the package leaflet available in Braille.

Choice of the appropriate medium should be made by the marketing authorisation holder in consultation with representatives of organizations for the blind and partially sighted. It is the responsibility of the marketing authorization holder to provide the package leaflet on request from patients’ organizations in an appropriate format and to ensure that the current version is supplied.

These requirements concerning the package leaflet for blind and partially-sighted persons also fully apply to parallel importers/distributors.

Chapter 3 Guidance concerning consultations with target patient groups for the package leaflet

1. INTRODUCTION

According to Articles 59(3) and 61(1) of Directive 2001/83/EC as amended by Directive 2004/27/EC new requirements apply to the package leaflet. Article 59(3) as amended requires that consultation with target patient groups ('user consultation') be carried out to demonstrate the readability and usefulness of the package leaflet to patients.

Article 59(3) reads:

> *"The package leaflet shall reflect the results of consultations with target patient groups to ensure that it is legible, clear and easy to use."*

Article 61(1) states that:

> "*The results of assessments carried out in cooperation with target patient groups shall also be provided to the competent authority.*"

Article 63(2) states that:

> *"The package leaflet must be written and designed to be clear and understandable, enabling the users to act appropriately, when necessary with the help of health professionals."*

In addition Article 28(2) and (3) of Directive 2001/83/EC requires that products authorised through the mutual recognition and decentralised procedures will result in a harmonised package leaflet between Member States.

2. SCOPE

For all marketing authorisations granted after 30 October 2005, all the requirements set out in Directive 2001/83/EC as amended apply. Therefore all package leaflets included in Community or national marketing authorisations have to be checked accordingly and the information about the patient consultation must be included in the application dossier.

For changes to existing marketing authorisations, the need for user consultation covers in principle situations where significant changes are made to the package leaflet, either through a variation or a procedure according to Article 61(3) of Directive 2001/83/EC.

3. FORMS OF PATIENT CONSULTATION

Articles 59(3) and 61(1) of Directive 2001/83 require that the package leaflet reflects the results of consultations with target patient groups to ensure that it is legible, clear and easy to use and that these results of assessments carried out in cooperation with target patient groups are also provided to the competent authority.

They do not define the precise method to be used. As a consequence, these provisions permit user testing as well as other appropriate forms of consultation.

3.1 User testing

One of the possible ways of complying with Article 59(3) is by performing a 'user testing' of the package leaflet.

User testing means to test the readability of a specimen with a group of selected test subjects. It is a development tool which is flexible and aims to identify whether or not the information as presented, conveys the correct messages to those who read it. Testing itself does not improve the quality of the information but it will indicate where there are problem areas which should be rectified. The user testing should be part of Module 1 of the application dossier.

Care should be taken that user testing is performed on the basis of the package leaflet as it is actually supplied with the product. This will require the use of a full mock-up of the leaflet in the colours and style and on the paper as used for the leaflet in the marketed pack. In particular, in the case of multilingual package leaflets, colour, style (including type size) and paper of the language version subject to user testing should be identical to the package leaflet as supplied with the marketed pack.

3.2 Other methods

Other methods than user testing may be acceptable provided that the outcome ensures that the information is legible, clear and easy to use so that patients can locate important information within the package leaflet, understand it and enables the user to act appropriately. Such alternative methodology will have to be justified by the applicant/marketing authorisation holder and will be considered on a case-by-case basis.

4. DEMONSTRATION OF PATIENT CONSULTATIONS

In general, performing the user testing or another justified consultation method will be essential prior to granting or varying any marketing authorisation under either the centralised, mutual recognition, decentralised or national procedures.

Member States and the European Medicines Agency agreed on harmonised Quality Review of Documents (QRD) templates for the package leaflet to ensure that the statutory information appears as intended by the Directive 2001/83/EC. Compliance with the QRD templates does not exempt from the obligation to undertake a user test or other form of user consultation.

4.1 New consultation for a medicinal product

In the following situations a user consultation is always required:

- First authorisation of a medicinal product with a new active substance,
- Medicinal products which have undergone a change in legal status,
- Medicinal products with a new presentation,

- Medicinal products with particular critical safety issues.

4.2 Reference to already approved package leaflets according to Article 59(3) and Article 61(1) of Directive 2001/83/EC

The evidence from tests on similar package leaflets may be used where appropriate. Examples of when this may be considered acceptable based on a sound justification by the applicant/marketing authorisation holder are:

- extensions for the same route of administration e.g. intravenous/intramuscular or oropharyngeal/laryngopharyngeal,
- same safety issues identified,
- same class of medicinal product.

It may be appropriate for an applicant/marketing authorisation holder to refer to a representative sample of package leaflets for medicinal products which comply with the new legislative requirements. The types of package leaflets should be chosen carefully to be representative of one or more of the following considerations:

- recently approved package leaflets for a corresponding medicinal product,
- reflect complex issues of risk communication which may need careful handling,
- medical terminology which requires detailed explanation .

However, certain package leaflets may require further user consultation to provide reassurance that patients will benefit from the information provided. This is e.g. the case where user consultation concentrates on one particular aspect of a leaflet which may need particular patient attention, e.g. expression of risk of side effects or complex instructions how to administer the medicinal product.

Member States, in the framework of the CMD(h), have issued additional guidance in the CMD(h)/QRD document “Consultation with Target Patient Groups – meeting the requirements of Article 59(3) without the need for a full test – Recommendations for Bridging”.

5. TESTING OF MULTIPLE LANGUAGE VERSIONS

The package leaflet should be legible, clear and easy to read in all EEA languages. As a matter of principle it is normally sufficient to undertake patient consultation in one EEA language. Results of such consultation should be presented in English for the centralised, decentralised and mutual recognition procedure, or in the national language for national procedures to permit the assessment of the test to be undertaken by competent authority responsible for granting the marketing authorisation.

In the centralised, decentralised and mutual recognition procedure, only the English language version of the package leaflet will be agreed during the scientific assessment.

The quality of translation should be the focus of a thorough review by the applicant/marketing authorisation holder once the original package leaflet has been properly tested and modified.

During the drafting of the original package leaflet every effort should be made to ensure that the package leaflet can be translated from the original to the various national languages in a clear and understandable way. It is important that the outcome of the user consultation is then correctly translated into the other languages. Strict literal translations from the original language may lead to package leaflets which contain unnatural phrases resulting in a package leaflet which is difficult for patients to understand. Therefore, different language versions of the same package leaflet should be 'faithful' translations allowing for regional translation flexibility, whilst maintaining the same core meaning.

Following the grant of the marketing authorisation, the responsibility for the production of faithful translations will rest with the marketing authorisation holder in consultation with the Member States/European Medicines Agency.

If user consultation has been performed on a package leaflet in the old QRD template, there is no need to be retested when updating according to the new QRD template.

6. PRESENTATION OF RESULTS

The presentation of results should be summarised[5] explaining how the consultation was executed and how the resulting package leaflet accommodated any need for change. The summary should be in Module 1.3.4 of the application and should have the following structure:

1. Product description
2. Consultation or test details, such as:
    · Method used
    · Explanation on the choice of population consulted
    · Language(s) tested
3. Questionnaire (including instructions and observation forms)
4. Original and revised package leaflets
5. Summary and discussion of results (subjects' answers, problems identified and revisions made to relevant package leaflet section)
6. Conclusion

All other details should be available on demand.

---

[5] Practical experience with presentation of results of consultations with target patient groups has shown that occasionally the data presented is not sufficiently detailed. Comprehensive data needs to be provided.

7. Approval by the competent authority

In approving package leaflets the competent authorities will look for evidence that people who are likely to rely on the package leaflet can understand it and act appropriately. Any consultation submitted in support of a package leaflet will need to cover the following:

- Data gathered from users under defined conditions
- The people who are likely to rely on the package leaflet for a particular medicine will depend upon a number of factors and may include carers (e.g. parents, partners, friends, as well as nursing assistants) rather than patients if the medicine is generally intended for administration by someone other than the patient.
- In order to ensure that those involved can understand and apply the information, the evidence presented must demonstrate that they can pick out the relevant information, interpret this and describe the action they would take as a result.
- The key information will need to be defined prior to the consultation by the marketing authorisation holder and is likely to include significant side effects, warnings, what the medicine is for and how to take/use the product.

Annex - Illustration - One way of undertaking a test of a package leaflet

This information is included for illustrative purposes only and is an example of a method that could be used for consultation with target patient groups.

The method described covers one-to-one, face-to-face, structured sets of interviews, involving at least 20 participants reflecting the population for whom the medicine is intended. As indicated above, other performance-based methods are equally valid, and competent authorities will judge applications on a case by case basis.

1. Performing the test

Testing of package leaflets may be done by the Marketing Authorisation holder or by a company contracted to carry out such testing on its behalf. It should be carried out by an experienced interviewer with good interview, observational and listening skills.

Ideally the person writing the package leaflet should help draw out the questionnaire and occasionally accompany the interviewer during testing, to enable direct transfer of learning. In addition, it may be useful to involve patient associations or 'expert patients' in the design of the test.

A full colour mock-up or specimen of the package leaflet intended for the market place must be used for testing.

2. Recruiting participants

Ensure a range of different types of people who are able to imagine needing to use the medicine. People selected should be representative of the population to be treated. For most medicines this criteria will be sufficient since the leaflet information will need to be accessible to all newly diagnosed patients. However, for some medicines you will need to involve carers.

Be sure to exclude people who are directly involved with medicines such as doctors, nurses and pharmacists.

Remember that information which can be used by the least able will be beneficial for all users. Try and include:

- particular age groups such as young people and older people – especially if the medicine is particularly relevant to their age group;
- new users or people who do not normally use medicines, particularly for information provided with new medicines likely to be used by a wide range of people (e.g. analgesics or antihistamines);
- people who do not use written documents in their working life;
- people who find written information difficult.

Recruit participants from wherever is most relevant and practical. For example you could use: older people's meeting points, self-help groups, patient support groups, community centres, parent and toddler groups.

3. SUGGESTED TESTING PROCEDURE

Only small numbers of participants are needed. The aim is to meet the success criteria in a total of 20 participants (excluding the pilot test). The important thing is not to re-test participants whom you have already tested. You can achieve this by undertaking:

- a pilot of around 3-6 participants is recommended to test that the questions will work in practice; as you gain experience, you may be able to use just two or three participants in the pilot test or move straight to the main testing phase;
- during testing review the results and make any necessary amendments to the package leaflet;
- repeat tests until you have satisfactory data from a group of 10 participants;
- a final test of a further 10 to see if the success criteria are also met in this further 10 (i.e. in 20 participants in total on the final proposed package leaflet).

4. PREPARING FOR THE TEST

You are advised to:

- draw up a new protocol for each medicine;
- include questions that reflect all the important and difficult issues, and use rigorous assessment criteria;
- make sure the questions cover finding, understanding and the participants ability to act appropriately;
- include a set of expected correct answers;
- design the test to last no more than 45 minutes, to avoid tiring participants.

Ensure that the questions reflect any specific issues for safe and effective use and compliance issues related to the medicine being tested. Testing is most beneficial when the questions relate to areas where patients' fears are greatest, such as side effects. Avoiding serious safety issues with a medicine during user testing of the package leaflet is not recommended.

The interviewer should:

- reassure the participants that it is the document which is being tested not them;
- allow the participant to read the whole of the leaflet if they wish;
- use a written set of questions for reference;

- ask the questions orally;
- adopt a conversational manner, allowing ample opportunity for interaction with the participant;
- ask participants, once they have located the required information, not to read it directly from the leaflet but to put it into their own words where appropriate.

As well as recording the answers to the questions, observe how each participant handles the leaflet and searches for information, noting, for example, whether people become lost or confused. This will yield valuable information about how to improve the structure of the package leaflet.

The questions should:

- adequately cover any critical safety issues with the medicine;
- be kept to a minimum; usually 12 -15 will be enough, though more may be required in special cases, e.g. if there are significant safety issues to be investigated;
- cover a balance of general and specific issues; a general issue might be what to do if a dose is missed, while a specific issue might relate to a side effect that occurs particularly with that medicine;
- be phrased differently from the text of the leaflet to avoid participants providing answers based merely on identifying groups of words;
- appear in a random order (i.e. not in the order the information appears in the leaflet);
- cover the preparation/handling instructions for products with complex administration devices; the use of dummy containers and active demonstration by participants is encouraged.

Copies of the protocol(s) including the questions asked, the responses offered, the interviewer's written observations and the different versions of the package leaflet tested must be submitted in module m-1-3-4 of the application dossier to the competent authority for review. Information on how to present the results is set out in Chapter 3, section 6.

5. SUCCESS CRITERIA

The purpose of user testing is to achieve a legible, clear and easy to use package leaflet and as such all suggestions from the user testing should be taken into consideration or otherwise justified. Questions asked within the test should be drafted carefully in order to test properly that key messages for safe use specific to the medicine can be understood and found within the text. Drafting easy or trivial questions simply with an aim of ensuring success must not occur.

A satisfactory test outcome for the method outlined above is when the information requested within the package leaflet can be found by 90% of test participants, of whom

90% can show that they understand it. That means to have 16 out of 20 participants able to find the information and answer each question correctly and act appropriately. However, it need not be the same 16 participants in each case. The success criteria will need to be achieved with each question. Results cannot be aggregated.

If you use a different performance based method, different success criteria may be appropriate. Competent authorities will consider these on a case-by-case basis.
